# Phylogenetic Analysis of West Nile Virus Isolates, Italy, 2008–2009

**DOI:** 10.3201/eid1705.101569

**Published:** 2011-05

**Authors:** Giada Rossini, Fabrizio Carletti, Licia Bordi, Francesca Cavrini, Paolo Gaibani, Maria P. Landini, Anna Pierro, Maria R. Capobianchi, Antonino Di Caro, Vittorio Sambri

**Affiliations:** Author affiliations: University of Bologna, Bologna, Italy (G. Rossini, F. Cavrini, P. Gaibani, M.P. Landini, A. Pierro, V. Sambri);; National Institute for Infectious Diseases “L. Spallanzani,” Rome, Italy (F. Carletti, L. Bordi, M.R. Capobianchi, A. Di Caro)

**Keywords:** Viruses, West Nile virus, flavivirus, vector-borne infections, phylogenetics, Italy, dispatch

## Abstract

To determine the lineage of West Nile virus that caused outbreaks in Italy in 2008 and 2009, several West Nile virus strains were isolated from human specimens and sequenced. On the basis of phylogenetic analyses, the strains isolated constitute a distinct group within the western Mediterranean cluster.

West Nile virus (WNV) is an arthropod-borne flavivirus affecting a wide range of vertebrates, including birds and mammals. The natural cycle of infection involves birds and mosquitoes (*1*); many species of wild birds act as amplifying hosts, whereas humans and horses are considered dead-end hosts (*2*). First identified in tropical Africa, WNV infection has been found in northern Africa, Israel, India, and Australia (*3*) and has progressively spread in the Americas since 1999 (*4**,**5*). WNV has been the cause of outbreaks and sporadic cases in central, eastern, and Mediterranean Europe for >45 years (*6*). Most strains responsible for the European and Mediterranean basin outbreaks are in lineage 1, with most grouped in the so-called European Mediterranean/Kenyan cluster (*7**,**8*).

In August 2008, an outbreak involving wild birds, horses, and humans affected 8 provinces in 3 regions in Italy (Emilia-Romagna, Veneto, Lombardy) (*9**,**10*). In 2009, a new epidemic was reported in the same region and in other neighboring regions in Italy, with up to 17 confirmed human cases of West Nile neuroinvasive disease (*11*).

## The Study

We obtained nucleotide sequences of the envelope (E) and nonstructural (NS)3/NS5 protein regions of 6 WNV human strains isolated during the 2008–2009 outbreaks in Italy and analyzed their phylogenetic relationships with other WNV strains isolated in Europe and the Mediterranean basin. Virus isolation on Vero E6 cells was performed from human serum samples obtained from patients with neuroinvasive WNV infection: 5 samples were collected during the 2009 outbreak from patients residing in the provinces of Ferrara (2 patients), Modena (1), and Mantua (2); 1 sample was collected during the 2008 outbreak from a patient living in Ferrara. Cytopathic effects were observed 6 days after the serum was injected into cell cultures; WNV infection was identified by immunofluorescence staining and confirmed by real-time reverse transcription–PCR (*9*).

Virus isolates were designed as follows: TOS-09 (Ferrara), NAL-09 (Ferrara), CHI-09 (Mantua), BAL-09 (Mantua), FAN-09 (Modena), and MAN-08 (Ferrara). After 1 passage, supernatants of infected tissue cultures were processed for viral RNA extraction and PCR amplification. Nucleotide sequences were obtained for the E gene (1,503 nt) and for a region of 5,759 nt encompassing the NS3, NS4a, NS4b, and NS5 genes, which are considered to be the most phylogenetic informative regions (*12**,**13*). Overlapping amplicons were amplified and bidirectionally sequenced. Primer sequences are available upon request.

**Figure 1:**
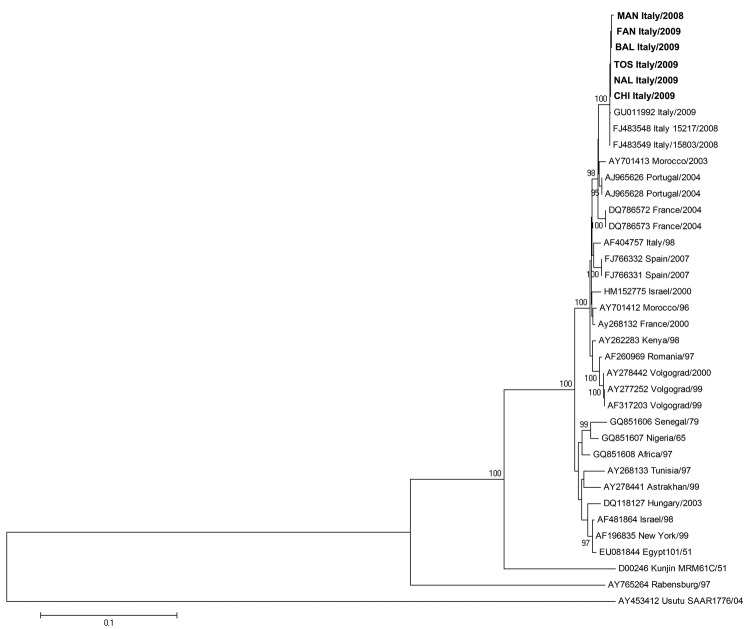
Phylogenetic trees of West Nile virus strains isolated during outbreaks in Italy, 2008–2009, based on nucleotide sequences of the complete envelope gene. Phylogenetic tree and distance matrices were constructed by using nucleotide alignment, the Kimura 2-parameter algorithm, and the neighbor-joining method implemented in MEGA version 4.1 (www.megasoftware.net/mega4/mega41.html). The tree was rooted by using Usutu virus as the outgroup virus. The robustness of branching patterns was tested by 1,000 bootstrap pseudoreplications. The percentage of successful bootstrap replicates is indicated at nodes, showing only values >80. The scale bar indicates nucleotide substitutions per site. Strains sequenced in this study are TOS-09, FAN-09, BAL-09, CHI-09, NAL-09, and MAN-08. Envelope nucleotide sequence GenBank accession nos: NAL-09, HM991272; TOS-09, HM991273; BAL-09, HM991274; CHI-09, HM991275; MAN-09, HM991276; FAN-09, HM991277. **Boldface** indicates the 6 newly sequenced strains.

**Figure 2:**
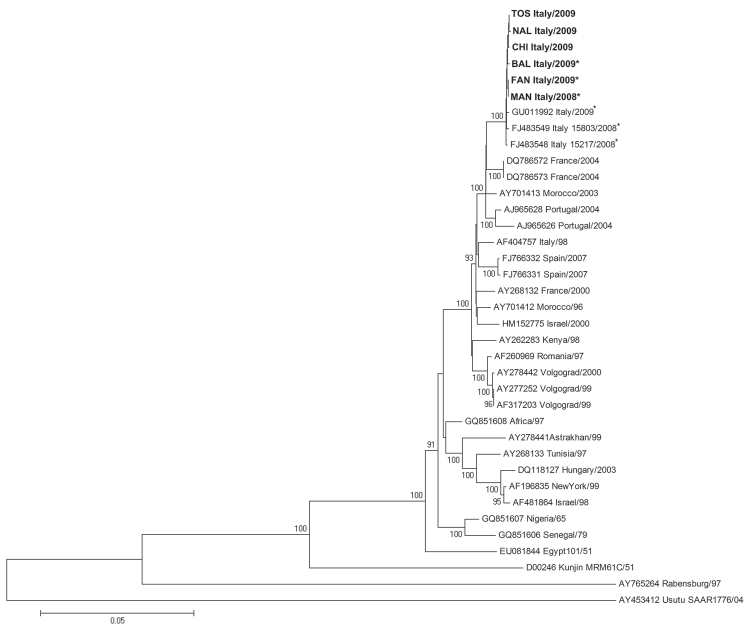
Genomic region encompassing nonstructural (NS) 3, NS4a, NS4b, and NS5 genes. Phylogenetic tree and distance matrices were constructed by using nucleotide alignment, the Kimura 2-parameter algorithm, and the neighbor-joining method implemented in MEGA version 4.1 (www.megasoftware.net/mega4/mega41.html). The tree was rooted by using Usutu virus as the outgroup virus. The robustness of branching patterns was tested by 1,000 bootstrap pseudoreplications. The percentage of successful bootstrap replicates is indicated at nodes, showing only values >80. The scale bar indicates nucleotide substitutions per site. Strains sequenced in this study are TOS-09, FAN-09, BAL-09, CHI-09, NAL-09 and 191 MAN-08. NS3/NS5 region nucleotide sequence GenBank accession nos.: NAL-09, HM641230; TOS-09, HM641225; BAL-09, HM641226; CHI-09, HM641227; MAN-09, HM641229; FAN-09, HM641228. **Boldface** indicates the 6 newly sequenced strains. *Strains from Italy carrying the T249P mutation.

Multiple alignments of the 6 new WNV sequences of E and NS3/NS5 regions, along with the corresponding sequences from 30 other WNV strains and isolates available in GenBank and Usutu virus as outgroup virus, were generated by using ClustalW software (www2.ebi.ac.uk/clustalw). Phylogenetic analyses of the E (Figure 1) and NS3/NS5 (Figure 2) sequences generated highly comparable topologies and confirmed that strains from Italy and all western Mediterranean strains belonging to lineage 1, clade 1a, were highly related. In addition, on the basis of the phylogenetic trees of E and NS3/NS5 sequences, we found that the 6 new sequences clustered with other strains isolated in Italy in 2008 and 2009, constituting a distinct and well-supported group within the western Mediterranean cluster (100% bootstrap on E and NS3/NS5 regions) (Figure1, Figure 2).

For the E gene, the mean genetic distance within the 6 new isolates from Italy was 0.08% at the nucleotide level, whereas the derived amino acid sequences were totally conserved. For the NS3/NS5 region, the mean genetic distance within the 6 new isolates from Italy was 0.10% at the nucleotide and amino acid levels.

Within the 2008–2009 cluster from Italy, the 6 newly sequenced human strains exhibited a 0.14% mean nucleotide distance with the 2 strains isolated in the Emilia Romagna region in 2008 from magpies (Italy 15803/08 and Italy 15217/08) and 0.15% with the first human WNV strain isolated in Italy in 2009 (Italy/09) in the Veneto region (*14*), in the E and NS3/NS5 genomic regions. On the basis of NS3/NS5 sequences, all strains from Italy isolated during the 2008–2009 outbreaks showed the highest relatedness to Morocco/03, France/04, and Portugal/04 with 1.08%, 1.28%, and 1.45% mean nucleotide distance, respectively, whereas they show a mean nucleotide distance of 1.56% with Italy/98. The close relatedness to Morocco/03, France/04, and Portugal/04 was also confirmed on E gene sequences (1.44%, 1.51%, and 1.17% mean nucleotide distance, respectively), compared with a mean nucleotide distance of 2.06% with Italy/98.

Comparison of the amino acid sequences of the E and NS3/NS5 region showed that all 2008–2009 strains from Italy exhibit a set of amino acid substitutions in the NS4a and NS5 proteins not shared with other western Mediterranean strains that could be considered as molecular signatures, e.g., I2209 and S2224 in the NS4a protein, A2786 and K2950 in the NS5 protein (Table A1). No relevant amino acid changes were observed in the E region.

Notably, the NS3 Thr249Pro substitution, suggested to be associated with increased virulence for American crows (*15*), was not uniformly present in all the 2008–2009 isolates in Italy. It was detected in all 2008 isolates, but heterogeneously distributed among the 2009 isolates. In particular, among the newly sequenced strains, P at NS3–249 was present in the BAL-09, FAN-09, and MAN-08 isolates, whereas T was present in the TOS-09, NAL-09, and CHI-09 isolates. Thus, this substitution could not be considered a hallmark of the well-defined 2008–2009 cluster in Italy.

## Conclusions

Although in the past decades several outbreaks of WNV infection have occurred in Europe and in the Mediterranean Basin (*6*), few viral isolates and nucleotide sequences are available from this geographic area, thus preventing an in-depth analysis of the molecular epidemiology of West Nile infection in the region. We provide 6 new nucleotide sequences of the E and NS3/NS5 regions from WNV strains isolated in 2008–2009 from patients in Italy with West Nile neuroinvasive disease and describe the isolates’ phylogenetic relationships with other WNV strains in the Western Mediterranean cluster.

The analysis of the E and NS3/NS5 sequences enabled a new phylogenetic reconstruction of the Western Mediterranean cluster, suggesting that the 2008–2009 strains from Italy constitute a distinct and well-supported group within the European–Mediterranean clade, characterized by specific amino acid changes in NS4a and NS5 proteins. The structure of phylogenetic trees, together with the presence of amino acid signatures, is consistent with a common origin of the strains circulating 2008–2009, supporting the concept of endemic circulation of WNV in Italy (*6*).

Furthermore, the T249P change in WNV-NS3 helicase has been suggested as increasing the virulence of WNV in birds in North America (*15*), although recent data do not support this hypothesis (*8*). Our results indicate that, at least in 2009 in Italy, there was a co-circulation of WNV strains carrying either a P or a T in this position. On the basis of our study, it is not possible to establish whether this was because of different virus introductions in Italy or reversion of some originally mutated strains. However, our findings do not support the hypothesis that the recent emergence of this mutation might have contributed to rapid spread of WNV infection with the occurrence of human cases in the Mediterranean Basin. More accurate studies are required to define the role of the T249P mutation in the pathogenesis and evolutionary history of WNV in recent outbreaks.

## Appendix

**Table A1:** Comparison of amino acid substitutions of West Nile virus strains isolated in Italy, 2008–2009, to different European and Mediterranean West Nile virus strains and to reference strain EG101*

AA position in WNV polyprotein	AA position in WNV protein	TOS‐09	NAL‐09	CHI‐09	BAL‐09	FAN‐09	MAN‐08	Italy 15803/08 (FJ483548)	Italy 15217/08 (FJ483549)	Italy‐09 (GU011992)	Italy/98 (AF404757)	Morocco/03 (AY701413)	France 405/04 (DQ786572)	Spain GE2o/V (FJ766332)	Portugal/04 (AJ965626)	EG101 (AF260968)
341	E‐51	A	A	A	A	A	A	A	A	A	A	A	A	T	A	A
378	E‐88	P	P	P	P	P	P	P	P	P	P	P	P	S	P	P
443	E‐153	G	G	G	G	G	G	G	G	G	G	G	R	G	G	G
602	E‐312	L	L	L	L	L	L	L	L	L	L	F	L	L	L	L
1754	NS3‐249	T	T	T	P	P	P	P	P	P	T	T	T	P	P	P
2209	NS4a‐85	I	I	I	I	I	I	I	I	I	V	V	V	V	V	V
2224	NS4a‐100	S	S	S	S	S	S	S	S	S	P	P	P	P	P	P
2265	NS4a‐141	M	M	M	L	M	M	M	M	M	M	M	M	M	M	L
2359	NS4b‐86	F	I	F	F	F	F	F	F	F	F	F	F	F	F	F
2516	NS4b‐243	I	I	I	T	T	T	T	T	T	T	T	T	T	T	T
2786	NS5‐258	A	A	A	A	A	A	A	A	A	V	V	V	V	V	V
2920	NS5‐292	P	P	P	P	S	S	S	S	S	S	S	S	S	S	R
2950	NS5‐422	K	K	K	K	K	K	K	K	K	R	R	R	R	R	R
3169	NS5‐641	T	T	T	T	A	A	T	T	T	T	T	T	T	T	T
3407	NS5‐879	I	I	I	H	I	I	I	I	I	I	I	I	I	I	I

*The amino acid position numbers refer to the EG101 strain. GenBank accession numbers are shown in parentheses. WNV, West Nile virus; E, envelope; NS, nonstructural.
